# Truncating a densely connected convolutional neural network with partial layer freezing and feature fusion for diagnosing COVID-19 from chest X-rays

**DOI:** 10.1016/j.mex.2021.101408

**Published:** 2021-06-05

**Authors:** Francis Jesmar P. Montalbo

**Affiliations:** Batangas State University

**Keywords:** Deep convolutional neural networks, COVID-19, Feature fusion, Medical image diagnosis, Image classification

## Abstract

Deep learning and computer vision revolutionized a new method to automate medical image diagnosis. However, to achieve reliable and state-of-the-art performance, vision-based models require high computing costs and robust datasets. Moreover, even with the conventional training methods, large vision-based models still involve lengthy epochs and costly disk consumptions that can entail difficulty during deployment due to the absence of high-end infrastructures. Therefore, this method modified the training approach on a vision-based model through layer truncation, partial layer freezing, and feature fusion. The proposed method was employed on a Densely Connected Convolutional Neural Network (CNN), the DenseNet model, to diagnose whether a Chest X-Ray (CXR) is well, has Pneumonia, or has COVID-19. From the results, the performance to parameter size ratio highlighted this method's effectiveness to train a DenseNet model with fewer parameters compared to traditionally trained state-of-the-art Deep CNN (DCNN) models, yet yield promising results.

• This novel method significantly reduced the model's parameter size without sacrificing much of its classification performance.
• The proposed method had better performance against some state-of-the-art Deep Convolutional Neural Network (DCNN) models that diagnosed samples of CXRs with COVID-19.
• The proposed method delivered a conveniently scalable, reproducible, and deployable DCNN model for most low-end devices.

## Graphical Abstract

**Figure:**
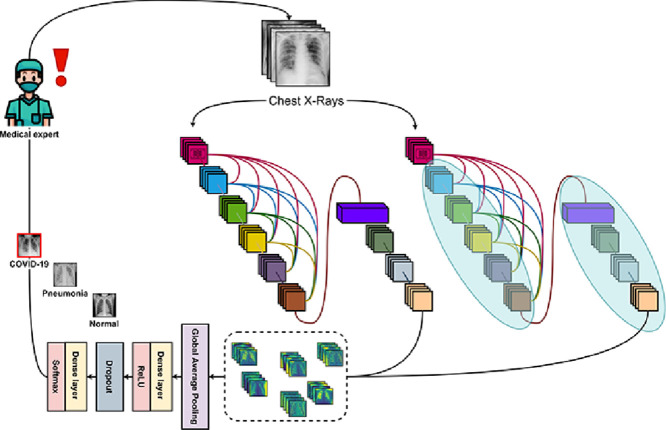

Specifications table

**Table:** 

Subject Area:	Computer Science
More specific subject area:	*Deep Convolutional Neural Networks and Medical Image Diagnosis*
Method name:	*A Non-Conventional Approach in Training a Deep Convolutional Neural Network based on Layer Truncation, Partial Layer Freezing, and Feature Fusion*
Name and reference of original method:	Not applicable – the proposed method relies on multiple approaches where the article had the following discussed and cited in the method section.
Resource availability:	**Source code and links to data used**: https://github.com/francismontalbo/fused-densenet-tiny

## Method Details

Deep Learning (DL) recently became one of the forefront solutions for conducting an automated medical image diagnosis of Chest X-Rays (CXR). However, with the sudden emergence of the recent Coronavirus, SARS-CoV-2, or specifically COVID-19, this automation process began to experience further struggles [1]. Compared to CXRs, diagnosing the COVID-19 virus from an individual using the real-time Reverse Polymerase Chain Reaction (rRT-PCR) provides better reliability. Unfortunately, though, such equipment can become costly and difficult to acquire. Only medical experts with specialized training can use it, making these requirements unavailable in most developing or less fortunate countries, having them rely on CXR diagnosis as an alternative [2]. Also, not all experts can immediately detect the presence of the new COVID-19 virus from CXRs as it becomes confusing due to its similarity with severe Pneumonia. Such a struggle had led to false results or late diagnoses. Through DL, specifically vision-based DL, researchers figured out a solution to employ such a method in automating the process to identify CXRs with the COVID-19 virus and distinguish it apart from someone with Pneumonia [3]. Though considered adequate, such a solution still inhibits some drawbacks as most proposed methods require the use of vast and complex models that require specialized equipment or infrastructure to operate.

Moreover, some models also tend to become unreproducible due to the lack of resources [4,5]. Therefore, this work proposed a solution that will maintain the reliability of large state-of-the-art models without the need for high-end devices or equipment to run and reproduce. With that said, developing countries and medical institutions lacking such resources can attain a faster and more accurate diagnosis of a patient's CXR infected by COVID-19 with less cost and effort.

**Fig. 1:**
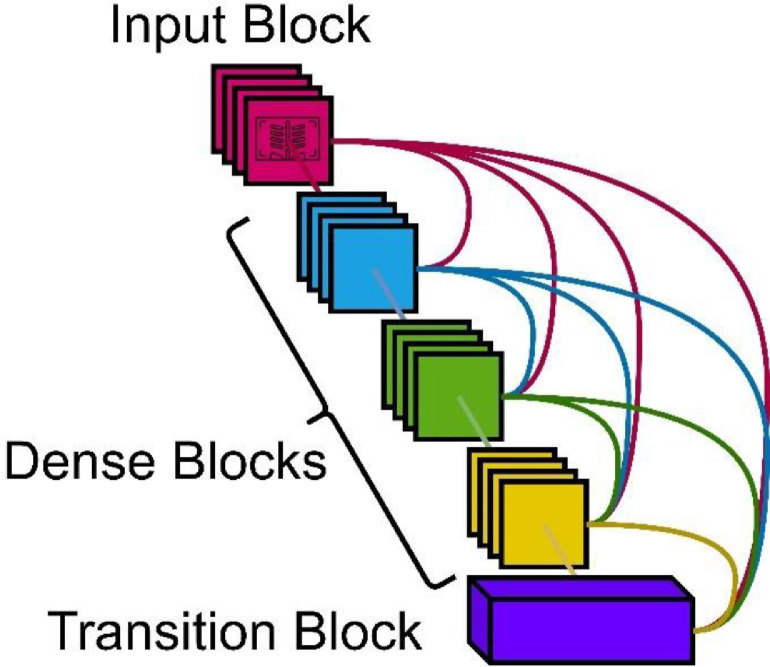
Visual Concept of a Densely Connected Convolutional Model.

The proposed method focuses on pre-training a DenseNet model through transfer learning (ImageNet weights) [6], truncation of its layers, replicating the truncated model, partial layer freezing its replicated version, and feature fusing both models. This work's base model focuses on the DenseNet121 model [7] that employed the mentioned methods to provide a lightweight yet a reliable model for diagnosing CXRs. Illustrated in Fig. 1, a DenseNet model's concept allows the propagation of features from every layer through concatenation. Such a design of the DenseNet resulted in lesser parameters compared to the summation method used in Residual Networks (ResNet) [8]. Findings also show that it reduced or somehow diminished the performance saturation problem with its architectural connectivity. As identified, training a DenseNet model even at greater depths maintained a significantly smaller parameter size than other recent state-of-the-art DCNNs [9].

**Fig. 2:**
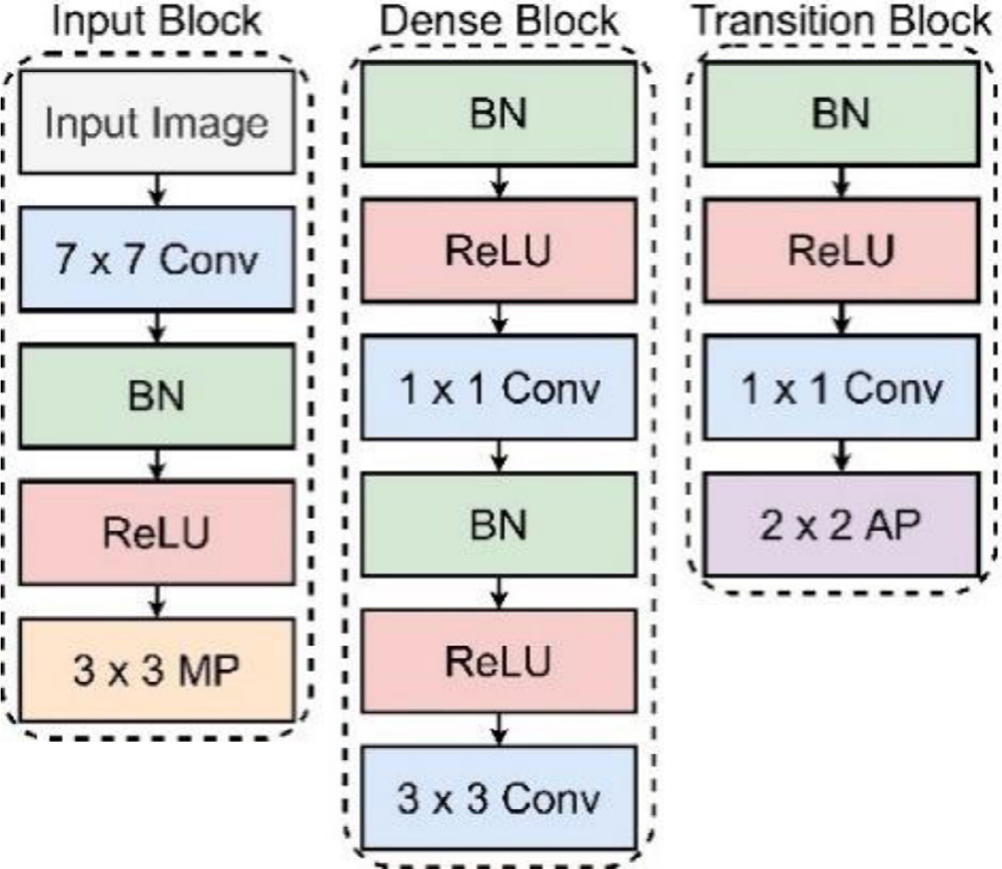
DenseNet blocks specifications.

Fig. 2 illustrates the input, dense, and transition blocks for a detailed specification of the model. The input block has a sequence of a 7 × 7 Convolution (Conv) → Batch Normalization (BN) → Rectified Linear Unit (ReLU) → 3 × 3 Max-Pooling (MP). The following block, called the dense block, has a sequence of BN → ReLU → 1 × 1 Conv → BN → ReLU → 3 × 3 Conv. For the last component, the transitional block follows a sequence of a BN → ReLU → 1 × 1 Conv → 2 × 2 Average Pooling (AP). The given set of sequences for each block generated significant improvements in feature extraction or generation compared to a less recent model like VGG [10] or GoogleNet [11]. The input block directly connects to a dense block, while the subsequent dense blocks tend to concatenate themselves with each other depending on the number of *k blocks* in the network*.* The transition block serves as the downsampling layer of the model where it contains a similar set of layers except for a 2 × 2 AP that reduces the feature size in half, which prevents the exhaustion of computing resources without much effect on its performance. The transition block has connections directly from the previous and succeeding dense blocks [9].

## Materials and Methods

### Chest X-Ray Dataset

Because of the support of large DL communities and other related fields, the curation of CXRs became more accessible for researchers. This work selected a readily prepared and reliable dataset from Sait et al. [12]. Their dataset contains various Joint Photographic Group (JPG) formatted CXRs segregated into three cases, a Normal CXR without any infections, a CXR infected with severe Pneumonia, and the other with COVID-19. The samples came from various reliable sources, therefore, having each class with a non-standard dimension.

Table 1 presents the dataset's specifications where the Normal CXR had 3270 samples, 4657 for Pneumonia, and 1281 for COVID-19. As observed, the dataset has an imbalanced characteristic due to the problematic acquisition of COVID-19 CXRs. Nonetheless, with the proposed method, such a problem will analyze if the imbalanced data will skew the model's performance towards the superior class. In training the model, the dataset had divisions of a train and validation sets where the train data for each class consists of 80% of its entire samples, having its remaining 20% used to validate each. Before the images entered the model to train, the images resized into 299 × 299 for a standardized training approach and prevented inconsistencies as some images have larger dimensions than the others.

**Table 1:** Chest X-Ray dataset specification.

Class label	Train (80%)	Validation (20%)	Total (100%)
Normal	2616	654	3270
COVID-19	1025	256	1281
Pneumonia (Bacterial and Viral)	3726	931	4657
**Total**	**7367**	**1841**	**9208**

### Truncation

For the first part of employing the proposed method, this work selected the DenseNet121 as the base model with its head eliminated, ending layers truncated, leaving the model only with its input block, fewer dense blocks of 9 connected with a transition block. The model only retained the mentioned layers leaving it without a dedicated output layer. The purpose of not having an output layer is to prepare its fusion with another to produce the fused version with a shared output layer that will efficiently handle the fused features. The fusion approach aims to increase the network's width and trainable parameters without elongating or expanding it.

**Fig. 3:**
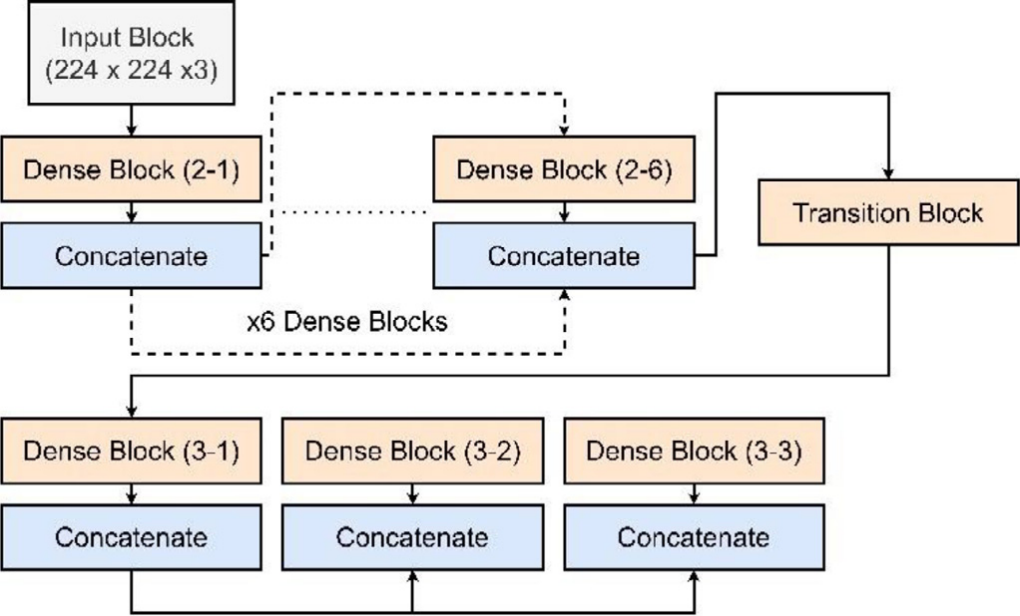
Layers and Connections of the Truncated DenseNet Model.

Fig. 3 illustrated the layers and connections of the proposed truncated DenseNet. The truncated version shows that the network consists of six dense blocks connected to a transition block followed by another set of 3 dense blocks. Unlike its larger DenseNet family counterparts, the proposed model significantly decreased its network size by 93%, where the base model DenseNet121 had about 8 million parameters, while the proposed truncated DenseNet model has only half a million.

Further, the truncation method significantly pertains to layer reduction, which decreases the parameters and overall weight size of a neural network model [13]. However, to utilize such an approach effectively, this proposed method identified a reasonable cut-point based on the number of features to preserve while maintaining a short end-to-end network and maintaining the original layer arrangement to prevent issues during transfer learning. As a result, the truncated model still attained 224 depth of features at the dense block 3-3. Also, the proposed truncation point maintained the entire entry block from the input block up to the dense block 2-6 concatenated to a transition block and another dense block from 3-1 to 3-3. Therefore, indicating that the proposed cut-point maintained the state-of-the-art core design of the DenseNet with a significantly lesser network yet a substantial feature depth, making it feasible to train via transfer learning and still conduct most of its initial feature extraction capabilities.

It is worth mentioning that the truncation initiates a shallower network to provide a faster propagation of weights during training and saves a significant fraction of computing cost [13]. However, in this work, the said advantage came with a significant disadvantage as well. Having fewer layers for feature generation or extraction reduced the model's performance due to fewer trainable parameters than a base DenseNet121 model. Hence, the proposed method included the approach of feature fusion.

### Mirroring and Feature Fusion

**Fig. 4:**
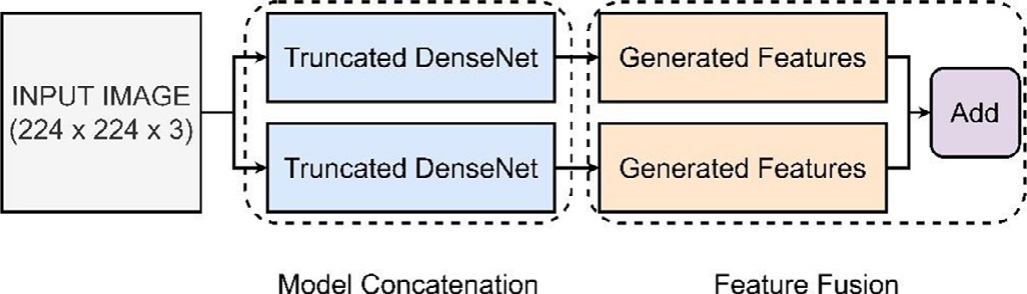
Feature Fusion and Fine-Tuning of the Truncated DenseNet Models.

After truncation, the model had significantly reduced in size, leading to lesser performance when learning features. For the model to increase its trainable patterns, the proposed method produced a mirrored version of the truncated DenseNet, as shown in Fig. 4. With a generated mirrored version of the truncated DenseNet, the models became concatenated into a single pipeline through an Add layer that expanded the range of features within the entire network. With the expanded feature depth and shorter network, the possibility of overfitting from the robust flow of generated features can occur. Thus, the employment of a proposed set of feature handling layers during fine-tuning counteracts overfitting issues.

The techniques employed can alleviate the problem of the reduced trainable parameters caused by the truncation approach. However, having similar models may only cause redundancy of generated features and an increased computing cost without much improvement. Therefore, the proposed method employed varying techniques to train each truncated model and provide feature diversity.

### Partial Layer Freezing and Fine-Tuning

With the potential scenario of redundant feature generation from the similar truncated DenseNet models during fusion, the proposed method employed partial layer freezing and fine-tuning techniques to alleviate such a problem. Beforehand, both models acquired readily available features from the ImageNet dataset through transfer learning to leverage their image recognition capability without prior training [14]. After the pre-training process, the models proceeded to partial layer freezing and fine-tuning to accommodate the selected tasks of classifying CXRs.

Partial layer freezing in the proposed method pertains to one of the models having its layers set to a frozen state, preventing the overwriting of the pre-trained weights from ImageNet during training and only updating its concatenation layer and the proposed set of ending layers. The concept of freezing layers came from fine-tuning that allows the model to adjust its pre-trained weights towards its newly replaced ending layers to solve specific tasks effectively. For example, in recent findings, fine-tuned models had proven to work better towards the classification of images than the random initialization approach and consumed shorter training durations that produced a robust set of features for a neural network and a classifier [15,16]. However, providing the same technique for the other model may generate similar outputs without feature contribution. Hence, in contrast, the other layer had layers set in a thawed state that allowed the flow of new weights and generated varied features through its entire network. Thus, with the combined approach of fine-tuning and re-initialization of new weights, the fused pipeline curated a broad spectrum of diverse features.

It is worth mentioning that the fusion approach also focused on producing a wide variety of features rather than just doubling the feature depth, 224 into 448. Thus, if the truncated models employed the same feature generation approach, it will only increase the feature depth but not entirely provide feature diversity. On the other hand, training both truncated models through random generation also falls into the same idea but may yield a far lesser performance due to the lack of features that the pre-trained weights provide. Therefore, instigating that random feature generation fused with pre-trained features from ImageNet integrated into a single pipeline can induce diversity and robustness for the model.

With the newly produced fused features, a set of tuning layers provides a counteractive approach to prevent potential overfitting and reduce misclassifications. The proposed layers consist of a Global Average Pooling (GAP) layer that replaces the traditional Fully Connected (FC) layers that averages the total pixel values of an image without deducting most of its feature importance. Compared to a standard flattened FC, the GAP also provides a better class interpretation for the neural network layers as it provides a corresponding feature towards a specific class [17]. Successively, an added Dense layer with an arbitrary number of 512 neurons provides additional trainable parameters. Activated by a Rectified Linear Unit (ReLU), the layer provides non-linearity and limits the output values into ones and zeros, leading to better efficiency due to an increased computation speed and lesser cost [18]. The other Dense layer activated by a softmax consists of only three neurons that correspond to the classes of interest, where the softmax acts as the multi-class logistic classifier of the model that assigns decimal values to each corresponding class. Thus, the diagnosis with the highest decimal value from the entire 100% becomes the diagnostic result. Finally, the proposed set of layers also has a Dropout layer with a rate of 0.5. The Dropout layer provides a stochastic reduction of neurons within the FC portion of the network, reducing overfitting and providing better regularization during training [19].

**Fig. 5:**
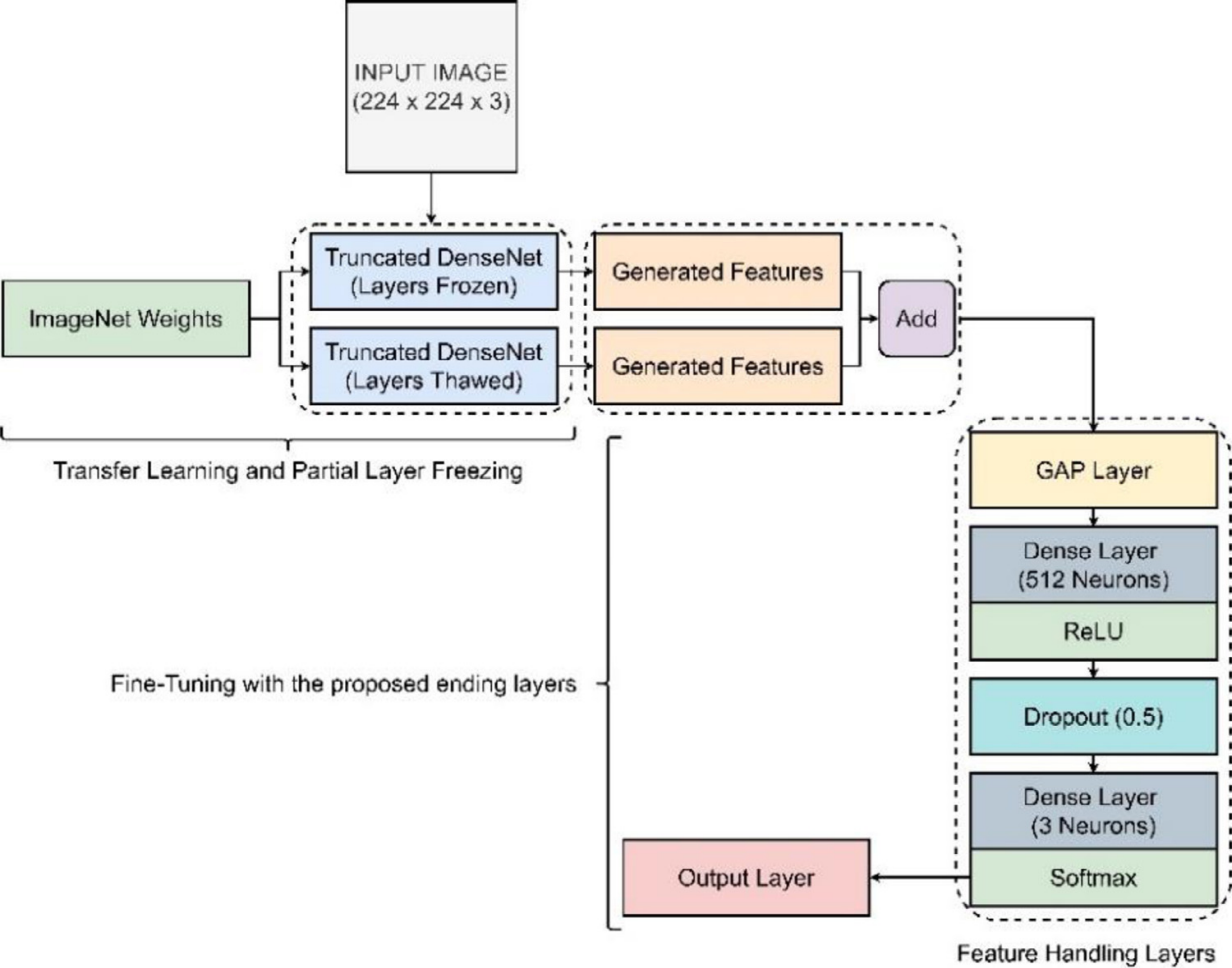
Pre-training, Partial Layer Freezing, and Fine-Tuning of the Fused Models.

Fig. 5 illustrates the discussed process of having the truncated models pre-trained with the ImageNet weights, partial layer freezing of the other half, and the initialization of the other's new weights.

### Model Compilation and Training

With the proposed method employed, the model does not train separately compared to an ensemble approach. Instead, it trained entirely as a whole, similarly to most DCNN models. During compilation, the model included a loss function and a set of configured hyper-parameters. For the production of results, the model employed an arbitrary and commonly selected value for each hyper-parameter. This approach highlights the proposed method to train in a more casual and less tedious approach without stringent optimizations yet produce competitive results against other state-of-the-art DCNNs. On the other hand, the accompanying loss function calculates and reduces errors during the training process.

As discussed, the task consists of three classes, Normal, COVID-19, and Pneumonia infected CXRs. This work selected to use the Categorical Cross-Entropy Loss (*CCE_Loss_*) as an appropriate loss function to accomplish the task, as it involves a multi-class classification rather than a binary classification [20]. In Eq. (1), the *CCE_Loss_* uses *M* as the classes with *c* instances from one to three, corresponding to the three CXR conditions. The calculation of loss differs from one class to another based on each observation *o* of the CXRs. The results of the prediction or classification *p* depend on the value of *y*.

(1) $$CCE_{loss}=-\sum_{c=1}^{M}y_{o,c}log\left(p_{o,c}\right)$$

Table 2 presents the following configured hyper-parameters: the optimizer, Learning Rate (LR), Batch Size (BS), and epochs. The optimizer provides an increased probability of attaining the lowest possible errors. This work selected the Adam optimizer as it consumes less memory than most optimizers and is starting to become a go-to optimization algorithm in most image classification tasks involving medical images [21,22]. Due to Adam's fast convergence capability, this work had set its LR to 0.0001, making it lower than a model that trains with a standard Stochastic Gradient Descent (SGD) [23]. The given LR can prevent heavy distortions that may cause the model to learn too fast and overfit during early training periods yet maintain a rapid convergence. A converged network usually entails that the model did not commit to overfitting or underfitting [24]. With the reduced network length due to the proposed method, the models managed to train rapidly even with a minimal BS of 16. Having a higher BS tends to train large models with robust datasets faster and induces slight performance improvements [25]. However, it does require tremendous amounts of memory and can deplete the resources early, wherein this work prevented such an event from occurring. With the Adam optimizer, an LR of 0.0001, and a BS 16, the selected epochs landed at 25, where it had shown the most efficient results based on training duration and performance. During the experiments, having more than the given epochs tends to cause lengthier training periods without performance improvements. On the other hand, training with fewer epochs limited the model to achieve higher accuracies.

**Table 2:** Hyper-parameter configuration.

Hyper-Parameter	Value
LR	0.0001
BS	16
Optimizer	Adam
DR	0.5
Epochs	25

## Results

### Evaluation metrics

To evaluate and compare the model's overall performance with the proposed method against other state-of-the-art models that trained conventionally, this work selected to use the standard metrics, including accuracy, precision, recall, and f1-score. The following metrics rely on the number of diagnosed CXRs distributed into the following descriptions, True Positive (TP), False Positive (FP), True Negative (TN), and True Positive (TP) [26]. TP refers to a correctly diagnosed CXR that has a specific infection, whether Pneumonia or COVID-19. TN, on the other hand, refers to a correctly diagnosed CXR without any infections as Normal. FP refers to a misdiagnosed Normal CXR with an existing infection, while FN presents the result the other way around. This work selected to use a confusion matrix to visualize the instances of diagnoses from the CXRs.

In the following equations below, the trained model with the proposed method had its overall accuracy, precision, recall, and f1-score calculated using the validation data.

(2) $$accuracy=\frac{TP+TN}{TP+TN+FP+FN}$$

(3) $$precision=\frac{TP}{TP+FP}$$

(4) $$recall=\frac{TP}{TP+TN}$$

(5) $$f_{1}-score=\frac{2*precision*recall}{precision+recall}$$

### Confusion Matrix

The confusion matrix graphically presents the diagnoses performed by the trained model. According to the results, the following images from the validation data had fallen into a specific category [27]. Therefore, each value that belongs to the diagonal palettes identifies a correctly classified image, whether TN (Normal) or TP (Pneumonia or COVID-19). Otherwise, it indicates that the model had made a misdiagnosis towards a specific CXR.

**Fig. 6:**
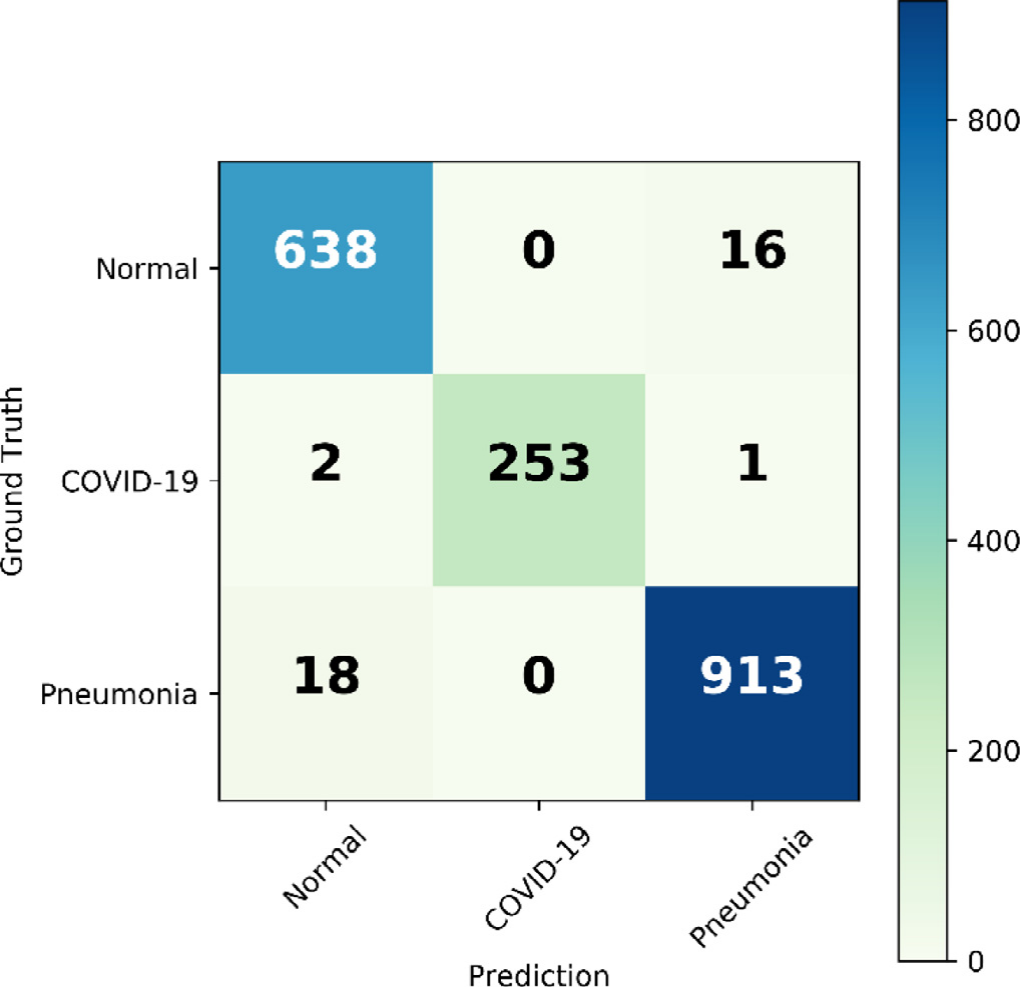
Results of diagnoses from the validation dataset using a confusion matrix.

Illustrated in Fig. 6, the model correctly diagnosed 638 Normal CXRs, 253 COVID-19 CXRs, and 913 Pneumonia CXRs. However, upon observations, the model committed misdiagnoses of 16 FPs of Normal CXRs, 2 FNs and 1 FP of COVID-19 CXRs, and 18 FNs of Pneumonia CXRs.

### Chest X-ray Diagnosis Performance

With the validation dataset diagnosed by the trained model, the equations provided can measure the model's performance towards a specific CXR class.

In Table 3, the trained model had acquired the best performance with COVID-19 with a 99.84% accuracy, followed by 98.10% and 98.04% from Pneumonia and Normal, respectively. Thus, even with the unbalanced sample size and class superiority of Pneumonia samples, the model still managed to attain unbiased results towards each class, having an indistinct result.

**Table 3:** Results of the overall performance.

Classes	Accuracy (%)	Precision	Recall	F_1_-score	Sample size
Normal	98.04	0.98	0.97	0.97	654
COVID-19	99.84	0.99	1.00	0.99	256
Pneumonia	98.10	0.98	0.98	0.98	931

## Discussion

Even with a truncated network, the DenseNet model managed to attain adequate trainable parameters to produce a wide range of features through transfer learning, feature fusing, partial layer freezing, and fine-tuning layers. Upon evaluation, the model's overall performance achieved 97.99% accuracy, 98.38% precision, 98.15% recall, and 98.26% f1-score. Justifying that even with the reduction of feature generators and model depth, the proposed method still trained the DenseNet model through a non-conventional method and attained competitive results.

Table 4 compares its performance against other state-of-the-art DCNNs trained through a conventional transfer learning and fine-tuning to validate the proposed method further. As presented, the proposed method did not achieve the highest scores compared to EfficientNetB0 and its base model, the DenseNet121. However, it outperformed most of the other well-known models that did not train with the proposed method.

**Table 4:** The comparison of performance with the proposed method against conventionally trained models

Model	Accuracy (%)	Precision (%)	Recall (%)	F1-Score (%)
DenseNet121 [7]	**98.48**	**98.71**	**98.59**	**98.48**
EfficientNetB0 [28]	98.21	98.59	98.18	98.39
Proposed Method	97.99	98.38	98.15	98.26
InceptionV3 [29]	97.99	98.31	98.23	98.26
ResNet152V2 [30]	97.88	98.25	98.09	98.17
Xception [31]	97.61	97.92	97.83	97.87
MobileNetV2 [32]	97.12	97.46	97.75	97.58
VGG16 [10]	96.58	97.06	96.94	96.97
InceptionResNetV2 [33]	96.14	94.48	96.90	95.59

The feature depth of only 224 from the proposed method limited the trained DenseNet model to attain higher results than its larger counterpart, the DenseNet121 model, which had 1024. Nonetheless, the proposed method's primary focus and aim significantly lean towards a lighter, easily reproducible, and deployable model for low-end devices in developing countries or medical facilities lacking the specialized equipment for the task. Further analysis and understanding of the proposed method can be found in [34].

**Fig. 7:**
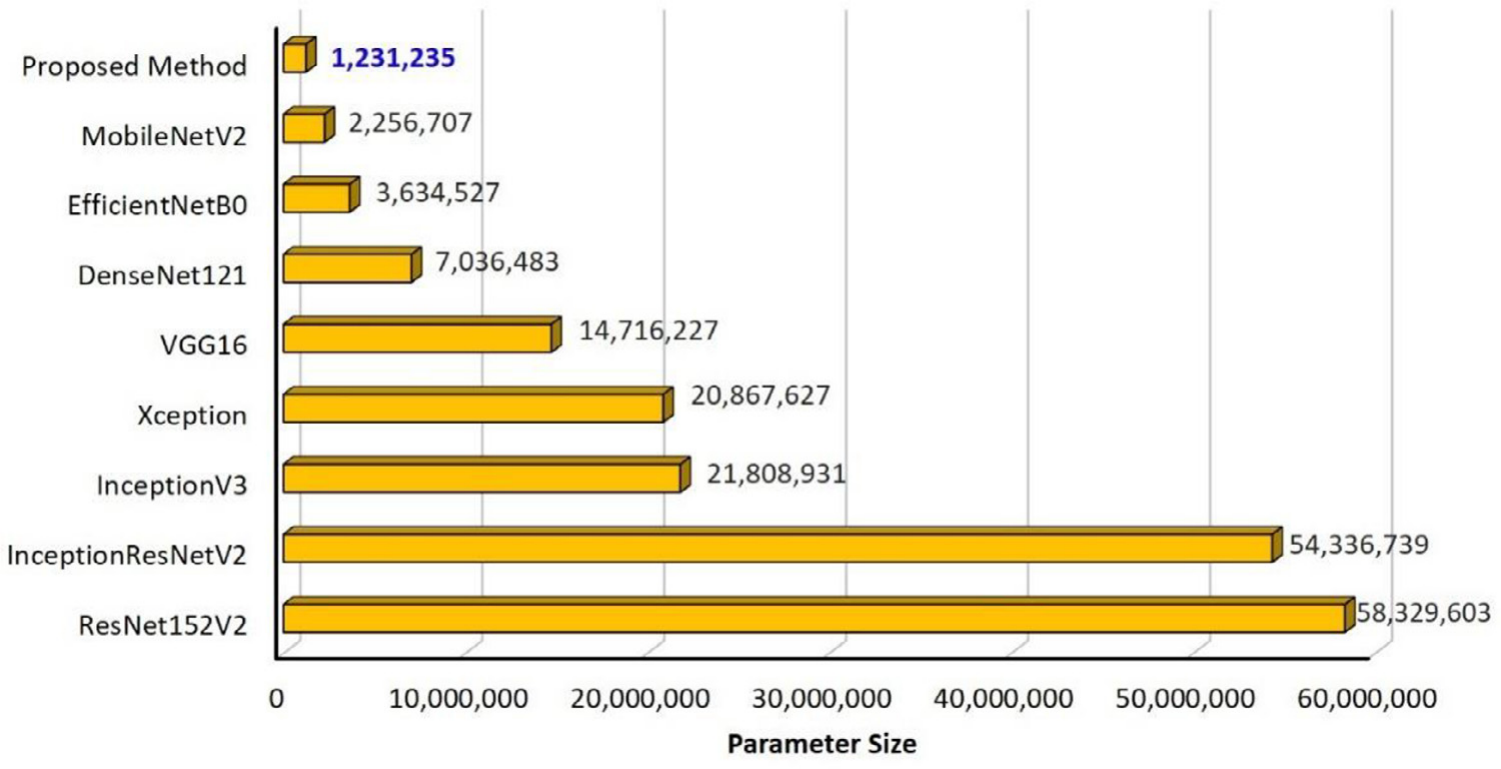
Comparison of parameter size with other state-of-the-art trained conventionally.

In Fig. 7, the DenseNet model trained with the proposed method outshined the entire set of DCNN models with the lowest number of parameters, exactly 1,231,235 million. Compared with the largest model, the ResNetV2-152, with a 97.88% accuracy and 58,329,603 million parameters, the DenseNet model trained with the proposed method achieved the best performance to parameter size ratio. With EfficientNetB0 and DenseNet121, even with a slight difference in performance, the proposed method still had a massive advantage with its lower number of parameters. With a minimal trade-off in performance, the lightweight yet reliable model can become easily deployable to possibly assist experts struggling to diagnose cases of CXRs with COVID-19 infections.

## Conclusions

In conclusion, though the method originated from various combined approaches, the entire process still elevated as a novel approach that trained a DCNN like the DenseNet model and diagnosed CXRs involving COVID-19 effectively and cost-efficiently. The variated and curated method had yielded remarkable performance compared to conventionally trained DCNNs with a significantly reduced parameter size. The slight trade-off still managed to pull through with a competitive performance yielded from the proposed method, justifying its effectivity towards CXR diagnosis involving COVID-19. Not only that the model works effectively, but it can also scale effortlessly. Re-training the DenseNet model with the proposed method with additional data will still consume less computing cost, training duration, and disk space than the larger models presented. Training models with a massive depth of layers will eventually require expensive equipment, long hours, and large disk capacities. However, this method's major drawback lies in its unorthodox preparation and lack of validation with other datasets.

Nonetheless, future researchers can reproduce this method through this article and conduct benchmark studies to test its full potential for other datasets and models. It is worth remembering that this method does not aim to replace other medical diagnostic methods. Instead, it aims to develop an effective and lightweight assistive tool for medical experts that may improve diagnosing COVID-19 in CXRs and other related conditions in the future.

## Declaration of Competing Interest

The Authors confirm that there are no conflicts of interest.
